# Fostering the Environmental Performance of Hotels in Pakistan: A Moderated Mediation Approach From the Perspective of Corporate Social Responsibility

**DOI:** 10.3389/fpsyg.2022.857906

**Published:** 2022-05-09

**Authors:** Bilal Ahmed, Hongming Xie, Malik Zia-Ud-Din, Muhammad Zaheer, Naveed Ahmad, Manman Guo

**Affiliations:** ^1^School of Management, Zhejiang University of Technology, Hangzhou, China; ^2^School of Management, Guangzhou University, Guangzhou, China; ^3^Faculty of Law, Islamia University of Bahawalpur, Bahawalpur, Pakistan; ^4^Faculty of Management, Virtual University of Pakistan, Lahore, Pakistan; ^5^Department of Business Administration, Lahore Leads University, Lahore, Pakistan

**Keywords:** corporate social responsibility (CSR), pro-environmental behavior (PEB), altruistic values, environmental performance (EP), hotel enterprises

## Abstract

The Islamic Republic of Pakistan has been a mere victim of climate change in recent years. The country needs emergency measures at every level to mitigate environmental dilapidation. The role of enterprises in the country’s environmental efforts is critical. In this regard, the hotel sector is known for its outsized carbon footprint. Knowing this, the current study aims to improve a hotel enterprise’s environmental performance (ENP) as an outcome of corporate social responsibility (CSR). The study also considers the mediating role of pro-environmental behavior (PEB) of employees and the moderating role of altruistic values (ALT). A hypothesized model was developed, which was validated by employing the structural equation modeling technique. The empirical results confirmed that CSR, directly and indirectly (through PEB), positively induces the ENP of a hotel enterprise. Whereas the conditional indirect role of ALT was also found significant. The study offers different implications for theory and practice, among which one important takeaway for the hotel sector is to realize the importance of employees to spur ENP of a hotel enterprise through their eco-friendly behavior. At the same time, the current work also advances the theory by highlighting the moderating role of ALT between the indirect relationship of CSR and ENP.

## Introduction

Perhaps, environmental protection is one of the top priority agenda items for every sector. Concern to preserve the environment is not new, however, the recent environmental issues like global warming, climate change, pollution, etc., have urged every stakeholder to partake in activities to preserve the environment (Kim et al., 2019). Almost every industry in the current ere notices the importance of sustainability, and thus, the concept does not remain an option for contemporary businesses but has emerged as a central business strategy (Ahmad et al., 2021a). Given that the environmental quality is determined, at least partially, by the interaction between human and the environment (Shafiei and Maleksaeidi, 2020; Kong et al., 2021), the importance of individuals’ sustainable behavior has been mounting in recent literature (Rustam et al., 2020; Chen et al., 2021). In an organizational context, the eco-friendly behavior, largely regarded as pro-environmental behavior (PEB), of employees is critical for any firm to improve its environmental footprint. Undoubtedly, the role of employees for a firm to achieve its different objectives is critical. The reason for such a view lies in the fact that employees have the practical knowledge and experience related to different operational areas in a firm, implying that a firm finds it difficult to achieve its goals without the considerable support of its employees. The view can also be seen in the work of Zientara and Zamojska (2018). From an environmental perspective, adopting PEB from employees provides extra support to a firm for achieving its sustainability objectives. At the same time, employees spend a sheer amount of their daily time, which again highlights the significance of their sustainable behavior (Yu et al., 2021).

A recent review of the literature pertinent to environmental studies unveils that most of the mainstream literature on PEB presents two perspectives—the consumer perspective (Miao and Wei, 2013; Chin et al., 2018; Chwialkowska et al., 2020) and the employee perspective (Lülfs and Hahn, 2013; Lu et al., 2017). To the extent of employees, research shows that different organizational and personal factors influence employees’ PEB. At the level of an organization, the role of corporate social responsibility (CSR) to influence the eco-friendly behavior of employees has received mounting importance quite recently (Afsar and Umrani, 2020; Murtaza et al., 2021; Shah et al., 2021). At the same time, the available literature also notes the importance of individual factors, such as values, to foster the behavior of employees (Hemingway, 2005; Osman-Gani et al., 2010).

Additionally, the available literature largely considered PEB as an outcome construct. For instance, the studies of Robertson and Carleton (2018) and Xu et al. (2021) are some recent relevant examples. However, there is a missing link in the literature: how a firm can foster its environmental performance by engaging its employees in PEB. Moreover, what could be the outcomes of PEB for a firm, is something that is an under-explored area. Against this backdrop, the current study aims to investigate the relationship between the CSR perception of employees and the environmental performance of a firm through PEB. At the same point, the current study also attempts to note the role of values, especially the altruistic values to influence the above-proposed relationship.

Given that the manufacturing industries directly impact the environment through their industrial practices, most of the environmental studies have focused on the manufacturing industries (Neppach et al., 2017; Martinez et al., 2018). However, the role of service industries was largely neglected previously. This is very recent that studies are referring to this sector from the perspective of the environment. The hotel industry is known for its outsized environmental footprint (Kasavan et al., 2019). Of more importance, a recent report shows that the contribution of this sector to the global carbon emission is 1% (Sustainable Hospitality Alliance, 2021), implying that this sector needs to reduce its environmental footprint significantly by improving its environmental performance. Responding to this issue, some global chains in this sector have significantly improved their environmental footprint. For example, the global player Hilton is committed to reducing its carbon intensity by 61% till 2030. Moreover, Hilton is also committed to reducing its water usage by 50% by 2030 (Hilton, 2021). The same holds in the case of Marriott International, another global hotel chain that has adopted several sustainable approaches to conserver natural resources (Marriott International). Despite such initiatives of some global players in the hotel sector, the majority of this sector still lags to enhance their environmental footprint. Specifically, when applied to the developing economies that are generally underperforming in terms of sustainability in each sector, the hotel sector is identified as a carbon-intense sector.

Pakistan, which is a developing economy, Where, pollution from different sectors, including the industrial sector, has been identified as a significant contribution toward such alarming environmental conditions. Country’s hotel sector has no exception, as it was also identified as a sector for its outsized carbon footprint. Waste creation, energy consumption, water, and food wastage are some of the major environmental issues associated with this sector in the country. This is why the current study has focused on this sector of the country to test the proposed relationships.

The current study intends to fill the knowledge gaps in three areas. First, the study attempts to fill the knowledge gap in the field of behavioral studies and environmental management literature from the perspective of CSR, employee behavior (PEB), and altruistic values under a unified model. To this end, the prior literature did not explore the potential of such constructs from an environmental perspective. Though, the role of altruistic values to guide the behavior of employees was discussed (King et al., 2017), the potential of altruistic values to shape eco-friendly behavior remained an under-explored area. Moreover, as already mentioned, most of the studies limit their potential by considering the PEB as an outcome construct, neglecting its potential importance to enhance the environmental performance of an enterprise. Though there exist few studies (Ahmad et al., 2021d; Naz et al., 2021), such studies are sparse.

Second, the consideration of the hotel sector also serves as an important contributor to advance the available literature, as a plethora of previous environmental studies were conducted in a manufacturing context (Neppach et al., 2017; Martinez et al., 2018), however considering the impact of the hotel sector on the environment, the current study focuses on services industries. Third, the current study also enriches the prior sustainability literature from the perspective of a developing economy. In this regard, it is to be mentioned here that in the past, the focus of environmental studies has been the developed countries, whereas the case of developing nations still witnesses an under-researched area.

The remainder of the current work is composed of four parts. The next part deals with the theory and related literature to develop the hypotheses and research framework. Similarly, the methodology part discusses the sample, data collection procedure, and instrumentation. The last two parts are dedicated to results and discussion, respectively. The results part deals with the statistical analysis and outcomes whereas, the discussion part discusses the study results in the light of previous findings. This part also discusses the theoretical and practical implications of the current study.

## Theory and Related Literature

The theoretical framework of the current research is rooted in the social identity theory developed by Tajfel (1978). Previous literature depicts that self-concept of a person is shown by his/her association with a specific social group (In this case, this social group can be regarded as the associated organization where employee is currently working). Various authors have employed this theory in studies related to human behavior (Chattopahyay and George, 2001; Nason et al., 2018; Ahmad et al., 2021b).

In the current context, a CSR inclined firm is expected to generate a value congruence between employees and a firm. This congruence of values led the employees to develop a strong bond with a socially responsible firm. All this process eventually constitutes a situation where employees are motivated to identify themselves with a responsible firm strongly. Altogether, the noble CSR engagement of a firm can significantly influence the behavior of employees positively due to the value congruence, which ultimately fosters the firm’s environmental performance. Moreover, the altruistic values of employees are also in congruence with the crux of CSR as both concepts (altruistic values and CSR) focus on the value of caring for others. Therefore, the current theoretical perspective finds social identity theory as a grounding theory to formulate different hypotheses.

### Corporate Social Responsibility and Environmental Performance

Corporations are assumed to operate without producing any negative environmental impact. Moreover, contemporary corporations are also required to assume their social responsibly while conducting different business operations (Adams and Zutshi, 2004). In the current era, a caring attitude from the businesses to preserve the environment is also demanded from different stakeholders, including consumers, NGOs, employees, and government (Kowalczyk and Kucharska, 2020). Responding to such pressures, firms incorporate different approaches, like CSR, to reduce their environmental footprint. It is pre-established in the literature that the CSR engagement of a firm can foster its environmental performance (Anser et al., 2020; Kraus et al., 2020). However, the mounting environmental issue around the globe has contributed toward a recent surge in the literature to relate CSR with environmental performance (Halme et al., 2020; Kraus et al., 2020). Referring to this, in their work Shabbir and Wisdom (2020) noted that CSR could spur the environmental performance of a firm in manufacturing industries. Other studies also acknowledge the importance of CSR to predict the environmental performance of an enterprise (Nazari et al., 2017; Halme et al., 2020). Research shows that CSR from the perspective of the environment is considered an important imperative to improving a business overall operational efficiency, including waste reduction, energy efficiency, water conservation, emission reduction, and others (Chuang and Huang, 2018). All such initiatives under the umbrella of CSR can logically be linked to the improved environmental performance of a firm. On a further note, CSR activities to preserve the environment are entirely volunteer engagement of firms beyond the state laws. Therefore, a firm under the philosophy of CSR goes an extra mile to preserve the environment. Hence, theoretically, it can be assumed:

**Hypothesis 1 (H1):** *Corporate social responsibility activities of a firm can positively induce its environmental performance.*

### Corporate Social Responsibility and Pro-environmental Behavior

To address different environmental challenges, firms consider different approaches. Among such approaches, promoting sustainable behavior among the employees has received mounting importance in recent literature (Byerly et al., 2018; Panno et al., 2018). Additionally, the latest Emissions Gap Report by United Nations Environmental Program (UNEP) notes that the eco-friendly behavior of individuals can significantly reduce environmental dilapidation (Unep, 2021). Specifically, it has been argued by different researchers that the CSR engagement of a firm can foster PEB among its employees (Suganthi, 2019; Raza et al., 2021). The underlying reason for this relationship lies in the caring attitude of a firm in the larger interest of society and the environment. Moreover, the CSR commitment of a firm is considered a voluntary effort for the betterment of the community. Referring to this, Dewhurst et al. (2009) mentioned that the volunteer nature of CSR is well placed to influence the extra-role performance of employees as compared to enhancing their bottom line (economic efficiency) performance. Because, PEB also falls in the lexicon of extra-roles, the CSR orientation of a firm can positively be linked with PEB of employees. Moreover, following social identity theory, the caring attitude of a socially responsible firm provides employees a logical reason to strongly identify them with their organization (a social group). This view from the perspective of social identity theory can be found in several studies (He et al., 2019; Lu et al., 2020; Raza et al., 2021). Moreover, the strong identity of employees with a social group, urge them to do everything for fostering the overall performance of their social group. All this process of identification not only improves employees’ formal behavior but also induces their informal behavior. In the current context, when employees observe the CSR engagement of their firm, they are self-motivated to support their social group, and thus they also adopt sustainable behavior at the workplace. Therefore, the following hypothesis is suggested.

**Hypothesis 2 (H2):** *Corporate social responsibility positively influences the pro-environmental behavior of employees.*

### Corporate Social Responsibility, Pro-environmental Behavior, and Environmental Performance

The seminal work of Podsakoff and MacKenzie (1997) indicated that employees’ extra-role behavior, especially their citizenship behavior, is central to a firm’s performance. They mentioned different reasons for this relationship, including employees’ supportive behavior to help other colleagues, learning new skills to support their firm, and showing responsible behavior. Moving forward, Walz and Niehoff (2000) acknowledged the role of employees to foster the performance of a restaurant. They further validated that the citizenship behavior of employees can lead a restaurant toward a better financial and quality performance. The study of Nielsen et al. (2009) also noted there exists a positive relationship between citizenship behavior and firm performance.

In the same vein, the positive association between the citizenship behavior of employees and the environmental performance of a firm has also been proposed by different scholars (Daily et al., 2009; Paillé et al., 2014). Relating to the current work theme, the work of Daily et al. (2009) argued that different behavioral intentions of employees related to the environment, for example, waste reduction and resource conservation, can lead a firm toward an improved level of environmental performance. More specifically, Roy et al. (2013) mentioned that the environment-specific behavior of employees could thrive a firm’s environmental performance.

When looked at from the perspective of the current study, it is quite possible to propose a mediating link of PEB between CSR and the environmental performance of a firm. Given that the firm’s CSR orientation helps employees build a strong identification with the firm, and by referring to social identity theory, employees put every effort to induce the overall performance of their firm, including the environmental performance. The above discussion clearly indicates that PEB directly influences the environmental performance of a firm and can also spur the relationship of CSR and environmental performance through its potential mediating role. Hence the following hypotheses may be proposed.

**Hypothesis 3 (H3):** *Pro-environmental behavior of employees positively influences the environmental performance of a firm.*

**Hypothesis 4 (H4):** *Pro-environmental behavior of employees mediates the relationship between corporate social responsibility and a firm’s environmental performance*.

### Moderating Role of Altruistic Values

Values are central to shaping individual behavior. As per the definition of Schwartz (1992), values are trans-situational goals that serve as a founding principle to guide individuals. Moreover, values are regarded as building blocks for developing the specific behavior at an individual level (Schminke et al., 2015). From an environmental perspective, values, specifically altruistic values, focus on the wellbeing of others collectively (i.e., considering the benefit of society and the biosphere) (Lee et al., 2014). Referring to the work of Stern and Dietz (1994), the authors are in agreement that the environmental values of employees can motivate them to act pro-environmentally. Moreover, values are assumed to be stable in nature, indicating the reason why several scholars stress the importance of values in shaping individual behavior. Indeed, it is also assumed that values hold a universal view (Milfont et al., 2010) and can predict the same individual behavior pattern in different cultures. Although the importance of values in influencing individual behavior has been significantly noted by different scholars in the available literature, however, given that values only provide a general foundation to develop a specific behavior. This is why, different researchers have stresses to note their indirect potential for behavior formation, rather than stressing on their direct potential. Hence, the moderating role of altruistic values has received a considerable attention from different scholars (Romani et al., 2013; Zasuwa, 2016). In the current settings, the CSR orientation of a firm inculcates the feelings of care for others in employees, which in turn urge them to act pro-environmentally. When the role of altruistic values of employees is also considered, it is expected that such relationship get strengthened to a further level. Therefore,

**Hypothesis 5 (H5):** *The presence of altruistic values moderates the mediated relationship between CSR, PEB, and environmental performance, such that the relationship is stronger in the presence of altruistic values.*

## Methodology

### Population, Sample, and the Data Collection

The targeted industry was the hotel sector of Pakistan which has been active in the country since its independence back in 1947. Given the country’s improved law and order situation during recent years, the hotel sector has witnessed considerable growth. From an economic perspective, the sector contributes 7% of the total GDP of Pakistan. Moreover, the sector is considered a labor-intensive industry, as currently employs almost 3.85 million people (Hadi, 2021). Despite the above encouraging statistics, the hotel industry is known for its out-sized environmental footprint (Islam et al., 2019). Considering the recent growth rate, this sector is expanding as more and more hotels join the sector each year. Accordingly, it is expected that this sector will continue its growth pattern in the future, implying that the expansion of this sector will also increase its environmental footprint. Presently, the hotel business in Pakistan includes different local, national, and international hotels, which exist in different cities. However, the country’s large cities hold the larger share of this sector compared to the small cities. International players like Avari, Marriot, Carlton, Regent, Hotel Mövenpick Karachi, Pearl Continental, and Ramada Plaza international, are also operating in different larger cities of the countries for many years. The cities like Karachi and Lahore in Pakistan are known for their industrial operations. This is why these cities are also regarded as the industrial hub in the country.

**FIGURE 1:**
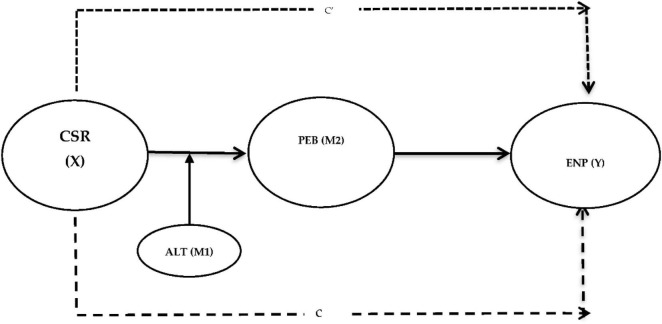
Research model of the current analysis: CSR (X) = the predictor variable, pro-environmental behavior—PEB (M2) = the mediating variable, Altruistic values—ALT (M1) = the moderating variable, Environmental performance—ENP (Y) = the criterion variable C = the effect of X on Y in the absence of M, c’ = effect of X on Y in the presence of M.

Of more importance, the above two cities are also known globally for a poor air quality index and environmental conditions. Especially, Lahore joins the list of the top five most polluted cities in the world (IQAir, 2021). Similarly, Karachi is also a bad victim of climate change which is a matter of public health of the rank and files. Both cities are also famous for the hotel sector, as almost every large national or international hotel chain exists in these cities. Given the poor environmental conditions in these two cities and the presence of large hotel players are factors that contribute to the consideration of these cities to test the proposed relations of the current study (Figure 1). The authors explored different hotels in these two cities which were known for their CSR engagements. To do this, the web pages of different hotels were observed by the authors in the first place, whereas in the second place, a personal visit was also paid by the authors in different hotels to know if they participate in any of CSR activities. Such exploration was helpful for the authors to identify hotels with CSR activities. It was observed that all upstream hotels were actively engaged in different CSR activities, and they were also communicating their CSR-related activities with different stakeholders, including employees. The authors contacted the concerned departments of the selected hotels for formal approval for data collection. The authors approached the hotels which responded to the authors positively on different dates from October 2020 to January 2021. The authors included different employees serving in different hotels in the sample. One can see Table 1 for more information on demographics.

**TABLE 1:** Demographic profile of the sample.

Demographic	Frequency (*n* = 511)	%
**Gender**		
Male	279	60.39
Female	183	39.61
**Age**		
18–25 years	49	10.61
26–30 years	101	21.86
31–35 years	153	33.12
36–40 years	98	21.21
Above 40 years	61	13.20
**Experience**		
1–3 years	82	17.75
4–6 years	179	38.74
7–9 year	141	30.52
10 years and beyond	60	12.99
**Category**		
Manager/supervisor	121	26.19
Non-Manager	341	73.81

A self-administered questionnaire (paper–pencil technique) was employed as a data-collecting instrument. Before finalizing such an instrument, the questionnaire items were assessed by experts in the field. Only after receiving valuable feedback from the experts, the final version of the questionnaire was provided to the survey respondents (Gjersing et al., 2010; Fernández-Gómez et al., 2020). The questionnaire was divided into two main parts. The first part was associated with the general demographic, and the second part included the main survey items on a seven-point Likert scale. The authors also observed the ethical guidelines of the Helsinki Declaration. In this regard, every respondent was given a separate informed consent form (attached with each questionnaire). Moreover, it was also conveyed to each respondent that quitting a survey at any stage was allowed if a respondent was uncomfortable in disclosing the required information. Moreover, the instrument did not include any information through which the anonymity of the respondent was at stake. Initially, 650 questionnaires were provided to different respondents, and eventually, 432 were collected back by the authors. Thus the true response rate was 66.46%.

### Measures

The current study constructs were operationalized by adapting different reliable and valid sources. Acknowledging the importance of pre-established scales from the perspective of reliability and validity (Hyman et al., 2006; Ahmad et al., 2021e; Murtaza et al., 2021), the authors felt the employed scales were quite suitable to serve the purpose of the current survey. In this vein, the famous CSR scale developed by Turker (2009) was considered by the authors. Indeed, this scale has been widely used by different scholars to operationalize the construct of CSR from the perception of employees. The studies of Tian and Robertson (2019) and Raza et al. (2021) are some ready examples. A sample item of the scale was “My hotel participates in activities, which aim to protect and improve the quality of the natural environment.” Twelve items were included to operationalize the construct of CSR. The Cronbach alpha value (α) in the case of CSR was 0.864. The construct of PEB was measured by employing the scale of Robertson and Barling (2013). This scale included a total of seven items, among which a sample item was “I turn lights off when not in use.” with an α value = 0.924. The scale to measure the construct of environmental performance—ENP was taken from the study of Kim et al. (2019). The original scale included seven items, however, the current study only considered five items as the dropped two items were pertinent to hotel reputation. A sample item was “Environmental management within our hotel has reduced waste.” The α value = 0.888 was found significant for this scale. Lastly, the items to measure the altruistic values were taken from the seminal work of De Groot and Steg (2007). There were eight items in this scale which the respondents were required to rate on an importance scale ranging from 1 to 7. The sample item included “as a guiding principle in my life, I consider pollution prevention.” The α value = 0.916 was observed for this scale.

## Results

### Detecting Common Method Variance

The issue of common method variance (CMV) has been regarded as a potential threat to contaminate a dataset. Especially, this potential threat becomes critical in cases where data collection for all constructs is made from a single source. More specifically, different researchers reported this issue as a contributing factor toward a false internal consistency (Podsakoff and Organ, 1986). The manifestation of CMV in survey data creates a situation in which response variance occurs due to a biased instrument, rather than happening due to the changes in perceptions of respondents (Chang et al., 2010). The occurrence of the potential threat of CMV leads an analyst to draw misleading results, implying that a whole research activity would be a wasted one.

Considering the importance of the above potential issue of CMV, and given that the data for the current study were collected from a single source, the authors opted to detect if the current study data encounters the issue of CMV.

A general approach toward detecting the CMV is to perform a single-factor test proposed by Harman (1976). To do this, the IBM-SPSS software was considered by the authors. The authors performed a single-factor analysis using this software without choosing any rotation type and fixing the number of factors to ‘1’. The results of this single factor analysis are given in Table 2 for the readers. The standard criterion to decide whether a dataset suffers from the issue of CMV is to see if the output of the single factor indicates a factor that explains the majority of the total variance (such that 50% or beyond). As one can see from the results given in Table 2, that the most variance explained by the single factor was 19.417, which was less than 50%. Thus, these results validate the non-existence of any CMV.

**TABLE 2:** Total variance explained by a single factor.

	Total variance explained					
Factor	Initial eigenvalues			Extraction sums of squared loadings		
	Total	% of Variance	Cumulative%	Total	% of Variance	Cumulative%
1	6.214	19.417	19.417	5.458	17.057	17.057
2	2.698	8.430	27.847			
3	1.698	5.306	33.153			
4	1.574	4.919	38.072			
5	1.344	4.201	42.273			
6	1.273	3.978	46.251			
7	1.244	3.888	50.140			
8	1.132	3.537	53.677			
9	1.106	3.457	57.134			
10	1.059	3.308	60.443			
11	1.016	3.174	63.617			
12	0.880	2.751	66.368			
13	0.814	2.544	68.912			
14	0.805	2.514	71.426			
15	0.742	2.320	73.746			
16	0.735	2.296	76.043			
17	0.716	2.239	78.281			
18	0.684	2.138	80.419			
19	0.641	2.005	82.424			
20	0.618	1.931	84.355			
21	0.556	1.737	86.091			
22	0.523	1.636	87.727			
23	0.505	1.578	89.305			
24	0.492	1.537	90.842			
25	0.477	1.490	92.332			
26	0.428	1.337	93.669			
27	0.416	1.299	94.968			
28	0.378	1.181	96.149			
29	0.353	1.103	97.252			
30	0.330	1.031	98.283			
31	0.296	0.925	99.208			
32	0.253	0.792	100.000			

*Extraction method = Principal Axis Factoring by fixing the number of factors to ‘1.’*

Given that the approach of single factor test is a very old technique, though still, it finds its place in different studies, the author decided to perform an advanced level test to detect CMV. In this respect, different authors have argued in favor of common latent factor test (CLT). To employ this technique, AMOS software was considered. In this regard, the authors developed a measurement model, which was then allowed to be influenced by a CLT. This common factor was assumed to directly influence each item of a construct. The constrained value of this common factor was fixed to ‘1.’ However, in line with the Harman single factor test findings, the CLT approach also indicated that the common factor is not explaining the sheer amount of total variance (50%). Thus these results cross-validated the findings of the single factor test in the early stage. Therefore, the CMV was not considered a potential threat to the dataset of the current study.

### Construct Evaluation: Factor Loadings, Validity, and the Reliability

The evidence against the presence of any CMV in the current dataset encouraged the authors to proceed with the further stages of data analysis. Hence the authors evaluated the constructs of the study by performing different statistical tests. For instance, the factor loading of each item was assessed to validate if each item’s loading is appropriate with respect to its corresponding construct. The common norm in this respect is to detect any item with a factor loading ideally less than 0.7 and to identify if there is a case of cross-loading with any item. These results are given in Table 3 for the readers. One can see from such results that no item showed a loading of λ < 0.70, implying that each item significantly loads to its respective factor. In like vein, the validity, especially the convergent validity, was also assessed by the author. For this reason, the average variance extracted (AVE) for each construct was calculated, then compared against the standard criterion of 0.50 (Kong et al., 2021). In this regard, if the AVE value for a construct is found less than 0.50, it means the construct is suffering from the issue of convergent validity. However, in all of the given cases, no such discrepancy was observed. For instance the AVE values for CSR, PEB, ALT and ENP were 0.64, 0.61, 0.73, and 0.64 respectively. Moreover, all the constructs were also evaluated for composite reliability (CR), which were found above the standard threshold level of 0.70 in every case (CSR—0.95, PEB—0.92, ALT—0.96, and ENP—0.90).

**TABLE 3:** Factor loadings, convergent validity, and composite reliability.

Items	Λ	λ^2^	*E*-Variance	Σλ^2^	Items	AVE	CR
My hotel participates in activities that aim to protect and improve the quality of the natural environment (CSR1)	0.74	0.55	0.45				
My hotel makes investments to create a better life for future generations (CSR2)	0.79	0.62	0.38				
My hotel implements special programs to minimize its negative impact on the natural environment (CSR3)	0.88	0.77	0.23				
My hotel targets sustainable growth, which considers to the future generations (CSR4)	0.71	0.50	0.50				
My hotel supports the non-governmental organizations that work in the problematic areas (CSR5)	0.92	0.85	0.15				
My hotel contributes to the campaigns and projects that promote the well-being of society (CSR6)	0.86	0.74	0.26				
My hotel encourages its employees to participate in voluntary activities (CSR7)	0.96	0.92	0.08				
My hotel’s policies encourage the employees to develop their skills and careers (CSR8)	0.77	0.59	0.41				
The management of my hotel is primarily concerned with the employees’ needs and wants (CSR9)	0.74	0.55	0.45				
My hotel implements flexible policies to provide a good work environment and life balance for its employees (CSR10)	0.72	0.52	0.48				
The managerial decisions related to the employees are usually fair (CSR11)	0.75	0.56	0.44				
My hotel supports employees who want to acquire additional education (CSR12)	0.73	0.53	0.47	7.71	12	0.64	0.95
I print double-sided whenever possible (PEB1)	0.72	0.52	0.48				
I put compostable items in the compost bin (PEB2)	0.7	0.49	0.51				
I bring reusable eating utensils to work (PEB3)	0.78	0.61	0.39				
I put recyclable material (e.g., cans, paper, bottles, batteries) in the recycling bins (PEB4)	0.85	0.72	0.28				
I turn lights off when not in use (PEB5)	0.74	0.55	0.45				
I take part in environmentally friendly programs (PEB6)	0.93	0.86	0.14				
I make suggestions about environmentally friendly practices to managers and/or environmental committees in an effort to increase my organization’s environmental performance (PEB7)	0.74	0.55	0.45	4.30	7	0.61	0.92
Unity with nature (ALT1)	0.91	0.83	0.17				
Preventing pollution (ALT2)	0.76	0.58	0.42				
Protecting the environment (ALVT3)	0.82	0.67	0.33				
Respecting the Earth (ALVT4)	0.92	0.85	0.15				
Social justice (ALVT5)	0.87	0.76	0.24				
A world at peace (ALT6)	0.89	0.79	0.21				
Helpful to others (ALT7)	0.87	0.76	0.24				
Equality (ALT8)	0.79	0.62	0.38	5.85	8	0.73	0.96
Waste reduction (ENP1)	0.81	0.66	0.34				
Energy conservation (ENP2)	0.74	0.55	0.45				
Less purchases of non-renewable materials (ENP3)	0.76	0.58	0.42				
Overall cos reduction (ENP4)	0.89	0.79	0.21				
Water conservation (ENP5)	0.82	0.67	0.33	3.25	5	0.64	0.90

*λ, item loadings; C.R, composite reliability; Σλ^2^, sum of square of item loadings; E-Variance, error variance.*

In the next stage of data analysis, a correlation analysis was performed (Table 4). This analysis was carried out to know the nature and value of correlation between different pairs of constructs. For example, the correlation test between CSR and ENP unveiled that this pair was positively correlated (*r* = 0.38), which provided initial support to H1 of the current study, implying that CSR and ENP move in the same direction with the changes in values. Meanwhile, the authors also performed another important validity test which was to check the discriminant validity of the study’s constructs. For this purpose, the square root value of AVE for each construct was first evaluated (bold values in Table 4). After calculating all such values, the authors compared them with the values of correlation. To further elucidate, the square root value of AVE for CSR was 0.80, and the correlation values were 0.36, 0.41, and 0.38. Generally, if the square root value of AVE of a construct exceeds the values of correlation (as in the current case), it is established that the criterion of discriminant validity for that construct is maintained. Lastly, the authors also developed different measurement models using AMOS, which were compared with the hypothesized model of the current study. It was revealed that the hypothesized model was the most significant model, implying that there was a good fit between theory and the data. These results are reported in Table 5.

**TABLE 4:** Correlations and discriminant validity.

Construct	CSR	PEB	ALT	ENP	Max-Min	Mean	*SD*
CSR	0.80	0.41**	0.36**	0.38**	7.00–1.00	5.33	0.63
PEB		0.78	0.46**	0.42**	7.00–2.00	5.68	0.55
ALT			0.86	0.33**	7.00–1.00	5.19	0.62
ENP				0.80	7.00–2.00	6.07	0.49

Sample size = 511; SD, standard deviation; **p-values < 0.05, diagonal values = discriminant validity values.

**TABLE 5:** Model fit comparison, alternate models vs. hypothesized model.

	Model-1 (Hypothesized)	Model-2 Two-Factor	Model-3 Three Factor
χ^2^ (*df*)	1783.692 (711)	2274.588 (526)	2068.396 (665)
χ^2^/*df*	2.51	4.32	3.11
NFI	0.94	0.78	0.89
CFI	0.96	0.79	0.92
RMSEA	0.049	0.072	0.050

### Hypotheses Testing

The current study employed the structural equation modeling technique (SEM) in AMOS software to test the hypothesized relationships. Indeed, SEM is an advanced-level data analysis technique that contemporary scholars largely prefer due to its ability to deal with complex models (as in the current study). Moreover, SEM can analyze the complex relationships (models with mediating and moderating constructs) simultaneously. This is one of the reasons why contemporary scholars are greatly inclined to use this data analysis technique (Cheah et al., 2020; Thakkar, 2020; Molnár et al., 2021; Puriwat and Tripopsakul, 2021). Moving forward, three Structural models were developed for hypotheses testing. Firstly, the authors developed the structural model to test only the direct relationships (H1, H2, and H3). This direct structural model did not include any moderating or mediating effect. The results of the direct effect structural model are reported in Table 6. As per the statistical results, the first three hypotheses of the current survey produce significant results as indicated by the positive beta values (β) and significant *p*-values (less than 0.05). These results statistically confirmed the theoretical statements given in hypotheses H1, H2, and H3.

**TABLE 6:** The results for hypotheses (H1, H2, and H3).

Path	Relation	Estimates	SE	CR	*p-*Value	ULCI	LLCI	Decision
CSR→ENP	+	(β1) 0.29**	0.022	13.18	***	0.462	0.377	Accepted
CSR→PEB	+	(β*2*) 0.37**	0.026	14.23	***	0.388	0.346	Accepted
PEB→ENP	+	(β*3*) 0.33**	0.034	09.71	***	0.411	0.372	Accepted

ULCI, upper-limit confidence interval; LLCI, lower-limit confidence interval. ** and ***, significant values.

Secondly, the structural model was developed to record the mediating effect of ALT between CSR and ENP (Table 7). In this vein, the bootstrapping option in AMOS was employed. A larger bootstrapping sample of 2000 was considered during this step as recommended by several extant researchers (Ahmad et al., 2021c,d; Guo et al., 2021). Moreover, a biased corrected 95% confidence interval was considered during this stage of the structural model. In this regard, the conventional approach of the Sobel-test for mediation analysis is considered an outdated approach due to its inferior power to explain the hypothesized relations compared to the bootstrapping method (Preacher and Hayes, 2008). The results of the structural model (mediated) at this stage unveiled the mediating effect of ALT, which was significant in the current case. Moreover, such results also indicated that ALT partially mediates between CSR and ENP as the beta value, which was 0.29 in the case of the direct effect model, was now reduced to 0.12 but still remained significant, implying that there exists a partial mediation effect of ALT between CSR and ENP. These results are indicated in favor of the statement of H4. Thirdly, the structural model was tested to evaluate the conditional indirect effect of ALT between the above proposed mediated relationship. The improvement in the beta value (β5 = 0.19) was indicative that the relationship was strengthened in the presence of ALT, implying that ALT moderates the mediated relationship of CSR and ENP. Therefore, H5 of the current study was also confirmed.

**TABLE 7:** Mediation and moderation results for H4, and H5.

Path	Estimates	*SE*	Z-score	*p-*value	ULCI	LLCI	Decision
CSR→PEB→ENP	(β*4*) 0.12**	0.019	6.31	***	0.353	0.341	Accepted
↓							
CSR→PEB→ENP	(β*5*) 0.19**	0.016	11.87	***	0.278	0.249	Accepted

ULCI, upper-limit confidence interval; LLCI, lower-limit confidence interval; SE, standard error. ** and ***, significant values.

## Discussion

The statistical findings of the current survey revealed the potential importance of CSR perceptions of hotel employees to foster the environmental performance of a hotel enterprise. The participation of a socially responsible hotel in different activities for the larger interest of society and the environment inculcates positive feelings among the employees. In return, the employees also participate in different environmental conservation activities, which induces the overall performance of a hotel enterprise from an environmental perspective. The CSR perspective of an enterprise to improve its environmental footprint is of utmost importance. Given that under the philosophy of CSR, a hotel enterprise continues its caring for others approach, and hence it puts every effort to preserve the natural resources, for instance, energy and water consumption, improving operations to generate waste to the minimum and facilitating the added support to the environment. Moreover, this CSR perspective also assumes to adopt eco-friendly activities, which exceeds the required state laws and considers a responsibility for any external negative effect on the part of an enterprise. At the same time, it has been reported that the environmental perspective of CSR deals with a series of actions taken by an enterprise to reduce its carbon footprint and thus to induce the overall environmental performance of an enterprise. These insights of the current analysis are also supported in the prior literature on environmental studies (Chuang and Huang, 2018; Portney, 2020; Mazurkiewicz, 2021). Specifically, the study of del Rosario Reyes-Santiago et al. (2019), showed that the proactive environmental strategies of a hotel can positively predict its environmental performance. Moreover, the work of Tan et al. (2017) posited that a better environmental strategy not only improves the environmental performance of an enterprise but also induces the overall financial performance. From the perspective of competition, the work of Skordoulis et al. (2020) mentioned that innovations pertinent to the environment can place an enterprise in a better competitive position compared to its rivals.

The current work also documents the role of employees in improving the environmental performance of a hotel enterprise as an outcome of CSR and PEB. In this vein, the CSR commitment of a hotel enterprise is well taken by its employees who are encouraged to support the environmental initiatives of their enterprise by performing different environmental improvement activities. Communicating the CSR concern of a hotel enterprise with the workforce is generally assumed to be helpful in improving the eco-friendly behavior of employees. A socially responsible hotel enterprise gives a clear message to its workforce that it has a concern for ‘caring of others. When employees see such a caring attitude, they are expected to regard this attitude positively. These results of the current work are also endorsed by different researchers who acknowledged the important role of CSR perception of employees in shaping environmental behavior (Suganthi, 2019; Tian and Robertson, 2019; Ahmad et al., 2021d). In this vein, the recent study of Abuelhassan and Elsayed (2020) acknowledged the role of employees in fostering the environmental performance of a hotel. The authors recommended that employees training, especially from the perspective of the environment, can positively spur the overall environmental performance of a hotel in the Egyptian context. In like manner, the work of Pham et al. (2020) also mentioned that green human resource management and the environmental performance of a hotel are positively related.

The current study also brings into discussion the potential moderating role of values, such as the altruistic values of employees to foster the environmental performance of an enterprise *via* CSR and PEB. Given that the role of values in shaping human behavior is critical, the moderating role of altruistic values provides additional support to the employees for their engagement in different environment-related behaviors. Because the focus of altruistic values is ‘caring for others and the CSR approach of an enterprise stresses the same, therefore a congruence of values between employees and the enterprise occurs (Afsar et al., 2018). This value congruence provides further strength to the employees to gauge their efforts toward their eco-friendly behavior, which eventually induces the environmental performance of a hotel through employees.

Moreover, the above discussion can also be extended from the perspective of social identity theory. Given that this theory explains to the employees to identify them with a social group, the CSR commitment of a hotel enterprise is one of such reasons due to which employees willingly identify themselves with a socially responsible enterprise. The literature argues that a strong social identity with a social group can lead the group members to show extra commitment to foster the overall group performance. From the perspective of the environment, employees strongly identify themselves with a socially responsible hotel enterprise, and thus they put every effort to support their social group by partaking in different activities to improve the environmental performance.

### Implications for Theory

The current work critically advances the available literature on CSR, employee behavior, and environmental studies. First, the current work is different from the available literature on PEB. The majority of the previous studies have considered PEB as an outcome variable (Suganthi, 2019; Raza et al., 2021), leaving the question unattended of how PEB can lead an enterprise toward enhanced environmental performance. Therefore, the current study extends the available literature on PEB from the perspective of environmental performance. A second important theoretical implication of the current study lies with the conditional indirect role of altruistic values to induce environmental performance through CSR and PEB. Though the moderating effect of values to shape employee behavior is already established (Marbach et al., 2019; Qazi et al., 2021), the current perspective in the domain of environmental management is not well explored. Yet another important theoretical implication of the current work lies with consideration of the service sector, especially the hotel sector of Pakistan. In this respect, most of the previous studies were carried out in an industrial setting from the perspective of manufacturing industries (Neppach et al., 2017; Martinez et al., 2018). However, the current study enriches the literature by highlighting the importance of the hotel sector to improve the environmental footprint of a country.

### Implications for Practice

At the same time, the study at hand also enriches the hotel sector by offering different practical implications. In this respect, the results of the current study can be observed by the policymakers in the hotel sector that how CSR can spur not only the environmental behavior of employees but also the overall environmental performance of a hotel. This finding is important for this sector, especially from an environmental perspective. In this vein, presently majority of hotels consider CSR from a philanthropic lens (charity, donation, etc.). The “Karighar” program by Serena is one example of this CSR perspective. However, this is the time for this sector to realize the potential role of CSR to reduce its outsized environmental footprint. The management needs to realize that the CSR perception of employees can significantly improve the environmental performance of a hotel enterprise. The approach of Monal hotel can be considered by its counterparts, as the hotel has significantly reduced its food wastage by constantly communicating its CSR role and highlighting the importance of food wastage. Importantly, such efforts were helpful to Monal for reducing its food wastage not only on the part of consumers but also on the part of employees. Therefore, the current study suggests the hotel sector closely aligns their CSR strategy from the environment perspective and the current philanthropic orientation. Because considering the philanthropic CSR efforts will be as good as “feeding the titanic with a teaspoon.”

Similarly, the current study’s findings also highlight the role of altruistic values for environmental improvement. In this regard, the hotel management is suggested to consider this factor during their recruitment and selection process. Moreover, for the current employees, the management needs to organize different seminars and workshops where employees can learn about environmental values to get themselves engaged in different environment-related causes at the workplace. Lastly, the negative environmental impacts of the hotel enterprises must be mitigated if these enterprises desire to be sustained in the future. In this respect, hotel management is required to improve their environmental footprint through different sustainability initiatives at an organizational level (i.e., green energy) and through a better environmental behavior of its employees for which a well-planned CSR strategy is a way forward for this sector.

### Limitations and Future Research Directions

Though the current work has made different important theoretical and practical implications, still it faces different limitations. However, such limitations also open new avenues for future researchers. In this vein, the first limitation lies with the geographic orientation of the current study, as this study only included two large cities (Lahore and Karachi). Therefore, it is desirable for future researchers to consider more cities so that a better and larger aspect may be realized. Secondly, the study only records the perceptual measures of CSR and environmental performance. Though such perceptual measures are helpful in a plethora of studies, using an objective measure of such constructs in the upcoming studies may generate more realistic outcomes. Similarly, the current study’s findings may remain limited in its scope because a cross-sectional survey design was employed, limiting the causality of association among different constructs. In this regard, a better approach for future studies may be to incorporate a longitudinal data design. Lastly, given that CSR is context and culturally specific, the current survey findings may remain similar for the like cultures (India, Bangladesh, etc.). However, due care is necessary in different cultures before interpreting the current survey results.

## Conclusion

Given that the concern for the environment has become the main topic of debate of the present time, all sectors are realizing the importance of sustainability. In this respect, rethinking sustainable practices in the hotel sector is becoming a matter of prime importance, as this sector is globally known for its outsized carbon footprint.

For promoting sustainability, the current study contributes by highlighting the importance of CSR to improve the environmental performance of a hotel through its employees. As this sector in the country constitutes a multi-million workforce, improving eco-friendly behavior among employees in this sector is of utmost importance to induce environmental performance. In this regard, organizational factors (CSR) and personal values (altruistic) are at heart to influence an employee’s behavior.

Based on the data collection from the employees of hotel industry, the important takeaway of the current research is that CSR activities of an enterprise create a value congruence between employees and the enterprise, which creates a strong bond on the part of employees to show extra commitment to a socially responsible enterprise, which then provides extra support to a hotel for enhanced environmental performance. Therefore, to have a sustainable future in this sector, undoubtedly, CSR is a way forward for hotel enterprises operating in Pakistan. Hotel industry needs to develop appropriate measures for promoting sustainable and eco-friendly practices. Hotels should introduce different CSR activities to promote pro-environmental behavior among employees and consumers.

The study results are insightful to analyze the adoption of sustainable practices in the hotel industry. However, there are some limitations associated with the current study. The data collected for the study were from the hotel industry and incorporated CSR, PEB, ALT, EVP, and introduced the mediating role of PEB. However, future studies might consider other mediating variables. Further, the study model can be applied to other sector for validation. One of the main limitations of the study is taking sample of study from two cities of the country, therefore, the generalizability is questionable. However, further studies might consider more cities to improve generalizibility.

## Data Availability Statement

The raw data supporting the conclusions of this article will be made available by the authors, without undue reservation.

## Author Contributions

BA: writing original draft. HX: reviewing and editing, and project sponsorship. MZ-U-D: literature review and data collection. MZ: data collection, project funding, reviewing, and comments. NA: data curation and data analysis. MG: proofread and editing and revision and editing.

## Conflict of Interest

The authors declare that the research was conducted in the absence of any commercial or financial relationships that could be construed as a potential conflict of interest.

## Publisher’s Note

All claims expressed in this article are solely those of the authors and do not necessarily represent those of their affiliated organizations, or those of the publisher, the editors and the reviewers. Any product that may be evaluated in this article, or claim that may be made by its manufacturer, is not guaranteed or endorsed by the publisher.
